# Two Novel C_3_N_4_ Phases: Structural, Mechanical and Electronic Properties

**DOI:** 10.3390/ma9060427

**Published:** 2016-05-30

**Authors:** Qingyang Fan, Changchun Chai, Qun Wei, Yintang Yang

**Affiliations:** 1 Key Laboratory of Ministry of Education for Wide Band-Gap Semiconductor Materials and Devices, School of Microelectronics, Xidian University, Xi’an 710071, China; ccchai@mail.xidian.edu.cn (C.C.); ytyang@xidian.edu.cn (Y.Y.); 2 School of Physics and Optoelectronic Engineering, Xidian University, Xi’an 710071, China; qunwei@xidian.edu.cn

**Keywords:** C_3_N_4_ allotropes, mechanical properties, electronic properties, superhard materials

## Abstract

We systematically studied the physical properties of a novel superhard (*t*-C_3_N_4_) and a novel hard (*m*-C_3_N_4_) C_3_N_4_ allotrope. Detailed theoretical studies of the structural properties, elastic properties, density of states, and mechanical properties of these two C_3_N_4_ phases were carried out using first-principles calculations. The calculated elastic constants and the hardness revealed that *t*-C_3_N_4_ is ultra-incompressible and superhard, with a high bulk modulus of 375 GPa and a high hardness of 80 GPa. *m*-C_3_N_4_ and *t*-C_3_N_4_ both exhibit large anisotropy with respect to Poisson’s ratio, shear modulus, and Young’s modulus. Moreover, *m*-C_3_N_4_ is a quasi-direct-bandgap semiconductor, with a band gap of 4.522 eV, and *t*-C_3_N_4_ is also a quasi-direct-band-gap semiconductor, with a band gap of 4.210 eV, with the HSE06 functional.

## 1. Introduction

Studies on light element-based materials trace back to the middle of the last century. Since Lavoisier found that diamond was isostructural to carbon and much denser than graphite, many studies have been devoted to its synthesis under high pressure [1,2,3,4]. More and more researchers have begun to investigate the carbon allotropes [5,6,7,8,9,10,11,12,13,14,15]. The second light element-based material to be evidenced was boron nitride. It includes three different structures: blende-, wurtzite- and graphitic-type structures. Cubic boron nitride (c-BN) was first elaborated upon in 1957 by Wentorf, who performed direct conversion using graphitic boron nitride (at 7 GPa and 1500 °C) [16]. Many boron nitride allotropes have been investigated by researchers, such as O-BN, Pbca-BN, Z-BN, W-BN, h-BN, bct-BN, P-BN, and cT8-BN. Interest in carbon nitrides has been initiated by studying materials that exhibit mechanical properties comparable with those of diamond. A fullerene is a molecule of carbon in the form of a hollow sphere, ellipsoid, tube, and many other shapes. Gueorguiev *et al.* [17,18] studied the formation mechanisms and structural features of fullerene-like carbon nitride (FL CNx), utilizing first-principles calculations.

Liu *et al.* first predicted *β*-C_3_N_4_ [19]; its structure originated in *β*-Si_3_N_4_, with carbon substituting for silicon. In the same way, *α*-C3N4 has been deduced from *α*-Si_3_N_4_, replacing silicon with carbon. The bulk of *α*-C_3_N_4_ and *β*-C_3_N_4_ is 387 and 427 GPa, respectively, which are slightly smaller than that of diamond (431 GPa [20]). Therefore, there are *sp*^2^ and *sp*^3^ hybridizations on carbon and nitrogen in *α*-C_3_N_4_ and *β*-C_3_N_4_, respectively. The *pseudocubic*-C_3_N_4_ structure is isostructural to *α*-CdIn_2_Se_4_ [21] and was first proposed by Liu and Wentzcovitch [22]. The network structure of *pseudocubic*-C_3_N_4_ consists of corners-linked CN_4_ tetrahedra in which the C-N-C angle is close to 109°, which ensures *sp*^3^ hybridization for nitrogen. The bulk modulus of *pseudocubic*-C_3_N_4_ is 448 GPa, which is slightly larger than that of diamond.

*Cubic*-C_3_N_4_ is another C_3_N_4_ phase and is isostructural to the high-pressure structure of Zn_2_SiO_4_, which was proposed by Teter and Hemley [23]. The structure of *cubic*-C_3_N_4_ is similar to that of *pseudocubic*-C_3_N_4_, including the hybridization. Mo *et al.* [24] and later Kroll [25] proposed a *γ*-C_3_N_4_ polymorph derived from a *γ*-Si_3_N_4_ spinel high-pressure structure. The largest difference between these structures involves the hybridization of nitrogen and carbon. In *pseudocubic*-C_3_N_4_ or *cubic*-C_3_N_4_, both carbon and nitrogen adopt *sp*^3^ hybridization. *Graphite* C_3_N_4_ (g-C_3_N_4_) consists of the stacking along the c-axis of graphitic planes. Teter and Hemley first described these graphitic planes as a hexagonal organization of C_3_N_3_ triazine cycles. Because of its graphitic structure, the bulk modulus is only 51 GPa [22,26].

We propose *m*-C_3_N_4_ (*m* denotes Monoclinic symmetry, space group: Cm) and *t*-C_3_N_4_ (*t* denotes Tetragonal symmetry, space group: *I*-42*m*), whose structures are based on *m*-Si_3_N_4_ and *t*-Si_3_N_4_ [27], respectively, with C substituting for Si. The mechanical and electronic properties of *m*-C_3_N_4_ and *t*-C_3_N_4_ are presented in this work.

## 2. Computational Method

Density functional theory (DFT) [28,29] calculations within Vanderbilt ultrasoft pseudopotentials [30] were performed using the Cambridge Serial Total Energy Package (CASTEP) code [31]. For the exchange and correlation functional, we used the Perdew–Burke–Ernzerhof (PBE) version of the generalized gradient approximation (GGA) [32]. For *α*-C_3_N_4_, *β*-C_3_N_4_, *d*-ZB-C_3_N_4_, *Pseudocubic*-C_3_N_4_, *Cubic*-C_3_N_4_, *g*-C_3_N_4_, *m*-C_3_N_4_ and *t*-C_3_N_4_, an energy cutoff of 520 eV was used for the wave functions expansion. High dense *k*-point [33] sampling, with a grid spacing of less than 2π × 0.025 Å^−1^ (7 × 17 × 9 for *m*-C_3_N_4_, 11 × 11 × 6 for *t*-C_3_N_4_, 12 × 12 × 12 for d-ZB-C_3_N_4_, 12 × 12 × 12 for *pseudocubic*-C_3_N_4_, 8 × 8 × 8 for *cubic*-C_3_N_4_, 10 × 10 × 6 for *g*-C_3_N_4_, 7 × 7 × 8 for *α*-C_3_N_4_, and 7 × 7 × 18 for *β*-C_3_N_4_) in the Brillouin zone, was used. The equilibrium crystal structures were achieved by utilizing geometry optimization in the Broyden–Fletcher–Goldfarb–Shanno (BFGS) [34] minimization scheme. The self-consistent convergence of the total energy was 5 × 10^−6^ eV/atom; the maximum force on the atom was 0.01 eV/Å, the maximum ionic displacement was within 5 × 10^−4^ Å, and the maximum stress was within 0.02 GPa. The electronic properties of *t*-C_3_N_4_ and *m*-C_3_N_4_ were calculated using the Heyd–Scuseria–Ernzerhof (HSE06) [35,36] hybrid functional.

## 3. Results and Discussion

### 3.1. Structural Properties

**Figure 1:**
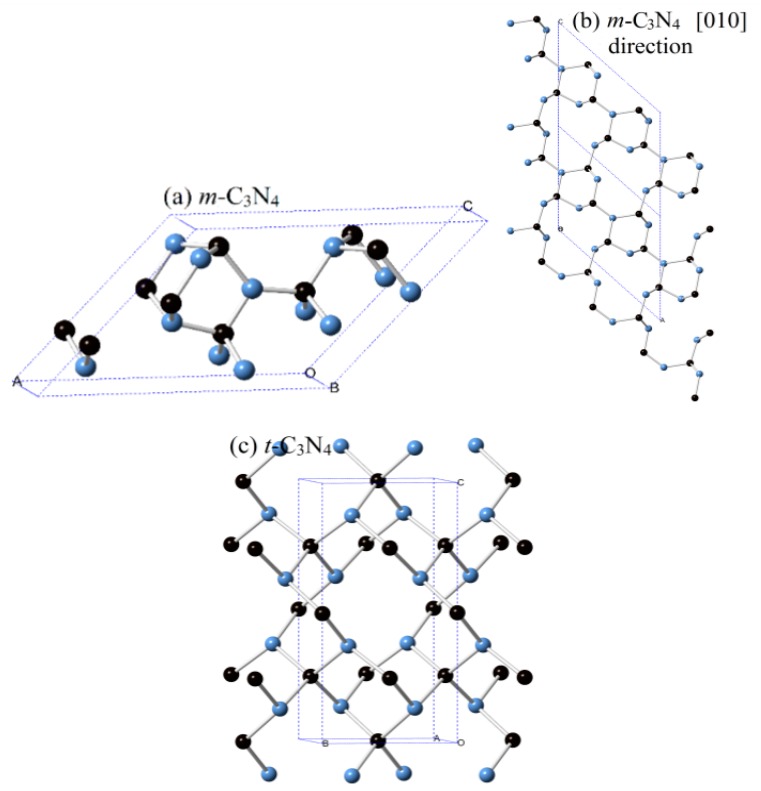
The crystal structures of: *m*-C_3_N_4_ (**a**,**b**); and *t*-C_3_N_4_ (**c**) (black spheres denote carbon atoms, blue spheres denote nitrogen atoms).

The crystal structures of *m*-C_3_N_4_ and *t*-C_3_N_4_ are shown in Figure 1. There are 14 (six carbon atoms and eight nitrogen atoms) atoms in a conventional cell of *m*-C_3_N_4_ and *t*-C_3_N_4_. Within this structure of *m*-C_3_N_4_, three inequivalent carbon atoms occupy the (0.8392, 0.0, 0.5479), (0.2917, 0.0, 0.8418) and (0.9864, 0.0, 0.3048) positions, and four inequivalent nitrogen atoms occupy the (0.2050, 0.0, 0.3856), (0.8843, 0.0, 0.8186), (0.3600, 0.0, 0.1253) and (0.5344, 0.5, 0.5771) positions, while for *t*-C_3_N_4_, two inequivalent carbon atoms occupy the (0.5, 0.5, 0.0) and (0.5, 0.0, 0.75) positions, and nitrogen atoms occupy the (0.7330, 0.2670, 0.8705) position, respectively. The basic building block of *m*-C_3_N_4_ is the six-membered zigzag carbon-nitrogen rings, which can be clearly observed in Figure 1a; the twelve-membered zigzag carbon-nitrogen rings in the [010] direction in the structure of *m*-C_3_N_4_ are shown in Figure 1b, while six-membered zigzag carbon-nitrogen rings and eight-membered gauche carbon-nitrogen rings exist in *t*-C_3_N_4_. The equilibrium lattice parameters of *m*-C_3_N_4_, *t*-C_3_N_4_, *d*-ZB-C_3_N_4_ (space group: *P*-43*m*), *cubic*-C_3_N_4_ (space group: *I*-43*d*), *pseudocubic*-C_3_N_4_ (space group: *P*-42*m*), *g*-C_3_N_4_ (space group: *P*-6*m*2), *α*-C_3_N_4_ (space group: *P*31*c*) and *β*-C_3_N_4_ (space group: *P*63*/m*) at ambient pressure are listed in Table 1. The calculated parameters of *α*-C_3_N_4_ and *β*-C_3_N_4_ are in excellent agreement with previous theoretical results (see Table 1).

**Figure 2:**
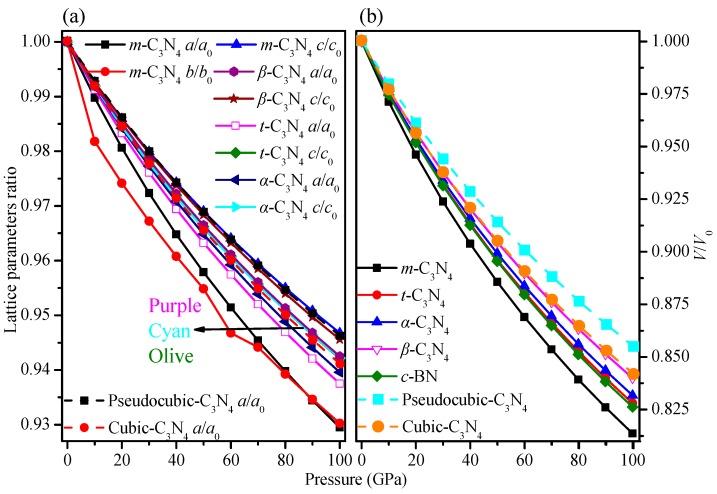
The lattice constants *a*/*a*_0_, *b*/*b*_0_, *c*/*c*_0_ and *V*/*V*_0_ of compression as functions of pressure and temperature for: *m*-C_3_N_4_ (**a**); and *t*-C_3_N_4_ (**b**).

The calculated pressure–volume relationships of *m*-C_3_N_4_ and *t*-C_3_N_4_, together with diamond, *c*-BN, and other C_3_N_4_ allotropes, are shown in Figure 2. The highest incompressibility along the *c*-axis is due to *m*-C_3_N_4_ in the C_3_N_4_ allotropes, while along the *c*-axis, *m*-C_3_N_4_ yields the lowest incompressibility at pressures from 0 to 87 GPa; along the *b*-axis, *m*-C_3_N_4_ yields the lowest incompressibility at pressures from 87 to 100 GPa. For the crystal structure, *pseudocubic*-C_3_N_4_ has the greatest incompressibility in the C_3_N_4_ allotropes discussed above, while *m*-C_3_N_4_ has the weakest incompressibility. However, the incompressibility of *t*-C_3_N_4_ is greater than that of *c*-BN and the incompressibility of *m*-C_3_N_4_ is weaker than that of *c*-BN.

### 3.2. Elastic Properties and Hardness

**Figure 3:**
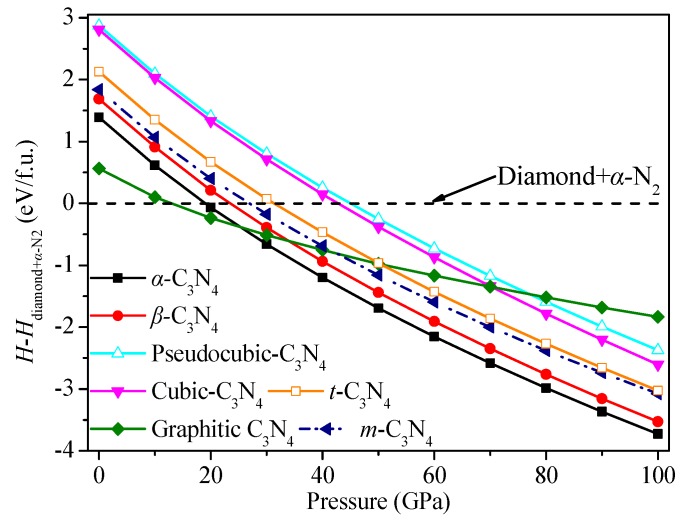
Calculated enthalpies of different C_3_N_4_ structures relative to diamond and *α*-N_2_ as a function of pressure.

In an effort to assess the thermodynamic stability of two novel C_3_N_4_ allotropes, enthalpy change curves with pressure for various structures were calculated, as presented in Figure 3. The dashed line represents the enthalpy of the summary of diamond and *α*-N_2_. It can be clearly seen that *g*-C_3_N_4_ has the lowest minimum value of enthalpy, which is in good agreement with previous reports and supports the reliability of our calculations [43]. *Pseudocubic*-C_3_N_4_ has the greatest minimum value of enthalpy. The minimum value of total energy per formula unit of *t*-C_3_N_4_ is slightly larger than that of *g*-C_3_N_4_, *α*-C_3_N_4_, *m*-C_3_N_4_, and *β*-C_3_N_4_ but much smaller than those of *pseudocubic*-C_3_N_4_ and *cubic*-C_3_N_4_, indicating that *t*-C_3_N_4_ and *m*-C_3_N_4_ should be thermodynamically metastable phases.

For monoclinic symmetry and tetragonal symmetry, there are different independent elastic constants. Monoclinic symmetry has thirty independent elastic constants (*C*_11_, *C*_22_, *C*_33_, *C*_44_, *C*_55_, *C*_66_, *C*_12_, *C*_13_, *C*_23_, *C*_15_, *C*_25_, *C*_35_ and *C*_46_), while tetragonal symmetry has six independent elastic constants (*C*_11_, *C*_33_, *C*_44_, *C*_66_, *C*_12_ and *C*_13_). The mechanical stability criteria of monoclinic symmetry are given by [44,45]:

(1) $$C_{ii}>0,i=1\sim 6.$$

(2) $$[C_{11}+C_{22}+C_{33}+2(C_{12}+C_{13}+C_{23})]>0$$

(3) $$(C_{33}C_{55}-C_{35}^{2})>0$$

(4) $$(C_{44}C_{66}-C_{46}^{2})>0$$

(5) $$(C_{22}+C_{33}-2C_{23})>0$$

(6) $$[C_{22}(C_{33}C_{55}-C_{35}^{2})+2C_{23}C_{25}C_{35}-C_{23}^{2}C_{55}-C_{25}^{2}C_{33}]>0$$

(7) $$\begin{array}{l} \Omega =2[C_{15}C_{25}(C_{33}C_{12}-C_{13}C_{23})+C_{15}C_{35}(C_{22}C_{13}-C_{12}C_{33})+\\ C_{25}C_{35}(C_{11}C_{23}-C_{12}C_{13})]-[C_{15}^{2}(C_{22}C_{33}-C_{23}^{2})+\\ C_{25}^{2}(C_{11}C_{33}-C_{13}^{2})+C_{35}^{2}(C_{11}C_{22}-C_{12}^{2})]+C_{55}g>0 \end{array}$$

(8) $$g=C_{11}C_{22}C_{33}-C_{11}C_{23}^{2}-C_{22}C_{13}^{2}-C_{33}C_{12}^{2}+2C_{12}C_{13}C_{23}$$

The criteria for the mechanical stability of tetragonal symmetry are given by [44]:

(9) $$C_{ii}>0,i=1,3,4,6.$$

(10) $$(C_{11}-C_{12})>0$$

(11) $$(C_{11}+C_{33}-2C_{13})>0$$

(12) $$[2(C_{11}+C_{12})+C_{33}+4C_{13}]>0$$

**Figure 4:**
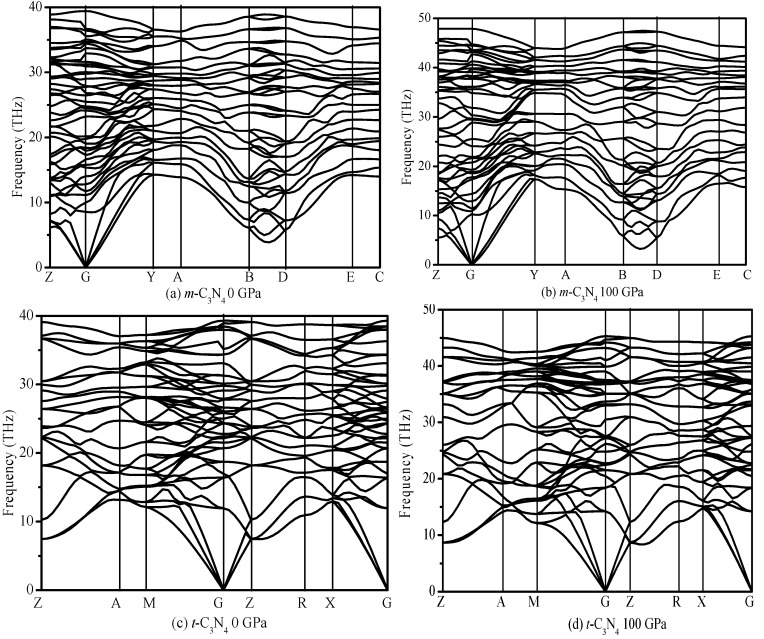
Phonon spectra for: *m*-C_3_N_4_ (**a**,**b**); and *t*-C_3_N_4_ (**c**,**d**).

The calculated elastic constants of *α*-C_3_N_4_, *β*-C_3_N_4_, *t*-C_3_N_4_, *d*-ZB-C_3_N_4_, *pseudocubic*-C_3_N_4_, *cubic*-C_3_N_4_ and *m*-C_3_N_4_ are listed in Table 2. Elastic constants under high pressure were also studied. The elastic constants under ambient pressure and high pressure of *t*-C_3_N_4_ and *m*-C_3_N_4_ satisfied the mechanical stability criteria of monoclinic symmetry and tetragonal symmetry. Namely, *t*-C_3_N_4_ and *m*-C_3_N_4_ are mechanically stable. To confirm the stability of *t*-C_3_N_4_ and *m*-C_3_N_4_, their dynamical stabilities should also be studied under ambient pressure and high pressures. Thus, we calculated the phonon spectra for *m*-C_3_N_4_ and *t*-C_3_N_4_ at 0 and 100 GPa, as shown in Figure 4. No imaginary frequencies are observed throughout the whole Brillouin zone, signaling dynamically the stabilities of *m*-C_3_N_4_ and *t*-C_3_N_4_. The calculated elastic modulus of *α*-C_3_N_4_, *β*-C_3_N_4_, *d*-ZB-C_3_N_4_, *pseudocubic*-C_3_N_4_, *cubic*-C_3_N_4_, *t*-C_3_N_4_ and *m*-C_3_N_4_ are listed in Table 3. Bulk modulus *B* and shear modulus *G* were calculated by using the Voigt–Reuss–Hill approximation [46,47,48]. The Voigt and Reuss approximation of monoclinic symmetry is calculated using the following equations [44]:

(13) $$B_{V}=\frac{1}{9}[C_{11}+C_{22}+C_{33}+2(C_{12}+C_{13}+C_{23})]$$

(14) $$B_{R}=\Omega {\left(a+b+c+d+e+f\right)}^{-1}$$

(15) $$a=(C_{33}C_{55}-C_{35}^{2})(C_{11}+C_{22}-2C_{12})$$

(16) $$b=(C_{23}C_{55}-C_{25}C_{35})(2C_{12}-2C_{11}-C_{23})$$

(17) $$c=(C_{13}C_{35}-C_{15}C_{33})(C_{15}-2C_{25})$$

(18) $$d=(C_{13}C_{55}-C_{15}C_{35})(2C_{12}+2C_{23}-C_{13}-2C_{22})$$

(19) $$e=(C_{13}C_{25}-C_{15}C_{23})(C_{25}-C_{15})$$

(20) $$\begin{array}{l} f=C_{11}(C_{22}C_{55}-C_{25}^{2})-C_{12}(C_{12}C_{55}-C_{15}C_{25})+\\ C_{15}(C_{12}C_{25}-C_{15}C_{22})+C_{25}(C_{23}C_{35}-C_{25}C_{33}) \end{array}$$

(21) $$G_{V}=\frac{1}{15}[C_{11}+C_{22}+C_{33}+3(C_{44}+C_{55}+C_{66})-(C_{12}+C_{13}+C_{23})]$$

(22) $$G_{R}=15\{\frac{4(f+h+i+j+k+l)}{\Omega }+{3[\frac{g}{\Omega }+\frac{\left(C_{44}+C_{66}\right)}{C_{44}C_{66}-C_{46}^{2}}]\}}^{-1}$$

(23) $$h=(C_{33}C_{55}-C_{35}^{2})(C_{11}+C_{22}+C_{12})$$

(24) $$i=(C_{23}C_{55}-C_{25}C_{35})(C_{11}-C_{12}-C_{23})$$

(25) $$j=(C_{13}C_{35}-C_{15}C_{33})(C_{15}+C_{25})$$

(26) $$k=(C_{13}C_{55}-C_{15}C_{35})(C_{22}-C_{23}-C_{12}-C_{13})$$

(27) $$l=(C_{13}C_{25}-C_{15}C_{23})(C_{15}-C_{25}).$$

The Voigt and Reuss approximation of tetragonal symmetry is calculated using the following equations [44]:

(28) $$B_{V}=\frac{1}{9}[4C_{13}+C_{33}+2(C_{11}+C_{12})]$$

(29) $$B_{R}=\frac{(C_{11}+C_{12})C_{33}-2C_{13}^{2}}{C_{11}+C_{12}+2C_{33}-4C_{13}}$$

(30) $$G_{V}=\frac{1}{30}[C_{11}+C_{12}+2C_{33}-4C_{13}+3C_{11}+12C_{44}+6C_{66}-3C_{12})]$$

(31) $$G_{R}=15\{\frac{18B_{V}}{(C_{11}+C_{12})C_{33}-2C_{13}^{2}}+{\left[\frac{6}{C_{11}-C_{12}}+\frac{6}{C_{44}}+\frac{3}{C_{66}}\right]\}}^{-1}.$$

The Hill approximation of monoclinic symmetry and tetragonal symmetry is calculated using the following equation:

(32) $$B_{H}=\frac{B_{V}+B_{R}}{2},G_{H}=\frac{G_{V}+G_{R}}{2}.$$

**Figure 5:**
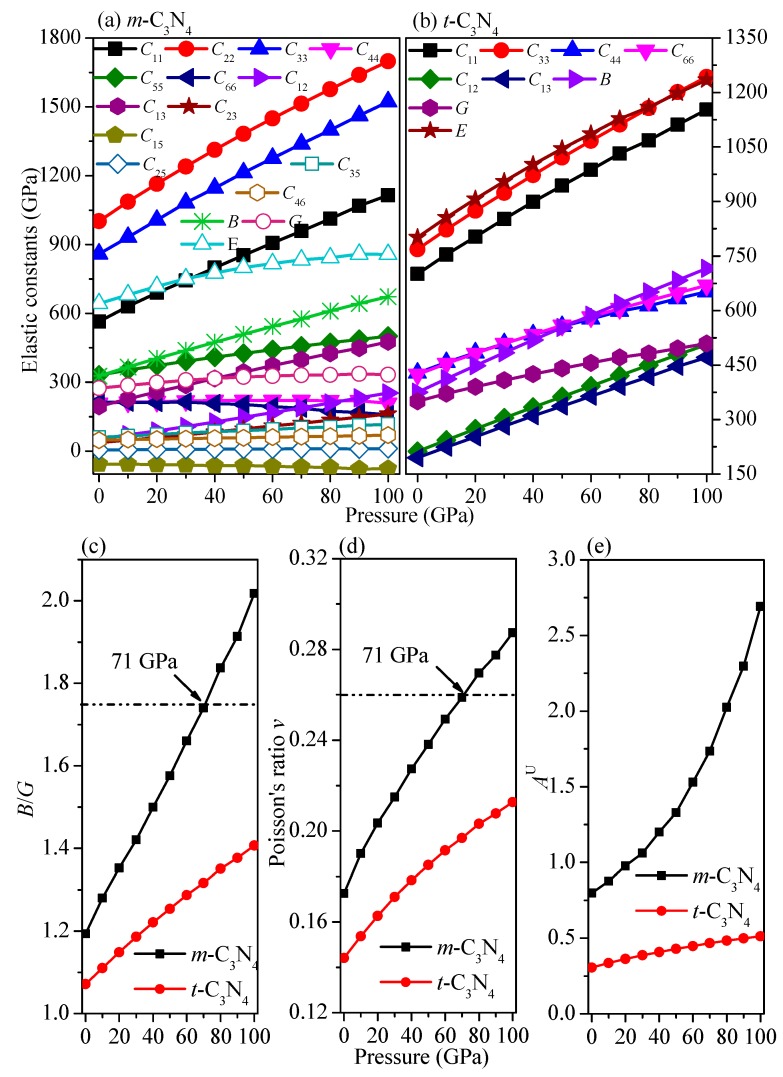
Elastic constants of *m*-C_3_N_4_ (**a**) and *t*-C_3_N_4_ (**b**) as a function of pressure, and the ratio *B*/*G* (**c**); Poisson’s ratio *v* (**d**); and *A*^U^ (**e**) of *m*-C_3_N_4_ and *t*-C_3_N_4_ as a function of pressure.

Young’s modulus and Poisson’s ratio can be calculated using the following formulas, respectively: *E* = 9*B_H_G_H_*/(3*B_H_* + *G_H_*), *v* = (3*B_H_* − 2*G_H_*)/(6*B_H_* + 2*G_H_*) [49]. The relationships between elastic constants and pressures are shown in Figure 5a,b. Most of them increase with pressure, whereas *C*_66_ and *C*_15_ of *m*-C_3_N_4_ decrease with pressure. The dependence of the elastic constants on pressure of *C*_22_ of *m*-C_3_N_4_, *i.e.*, *dC*_22_/*dP* = 6.97, means that *C*_22_ of *m*-C_3_N_4_ increases fastest among all elastic constants.

The dependence of bulk modulus, shear modulus and Young’s modulus on pressure of *m*-C_3_N_4_ and *t*-C_3_N_4_ is 3.49, 0.59, and 2.15 and 3.41, 1.59, and 4.35, respectively. Young’s modulus of *t*-C_3_N_4_ increases faster than other elastic modulus, while the increase in the shear modulus of *m*-C_3_N_4_ is the slowest. At ambient pressure, the bulk modulus of *α*-C_3_N_4_, *β*-C_3_N_4_, *m*-C_3_N_4_ and *t*-C_3_N_4_ are 387 GPa, 406 GPa, 327 GPa and 375 GPa, respectively. The calculated hardness of *α*-C_3_N_4_, *β*-C_3_N_4_, *m*-C_3_N_4_, *t*-C_3_N_4_, *Cubic*-C_3_N_4_, *d*-ZB-C_3_N_4_, Pseudocubic-C_3_N_4_ and *c*-BN are shown in Table 3. The bulk modulus of *t*-C_3_N_4_ is 375 GPa, which is slightly larger than that of *c*-BN, while the bulk modulus of *m*-C_3_N_4_ is slightly smaller than that of *c*-BN. The hardness of *m*-C_3_N_4_ is only 37 GPa, which is approximately half of that of *α*-C_3_N_4_, *β*-C_3_N_4_, *d*-ZB-C_3_N_4_, pseudocubic-C_3_N_4_ and *t*-C_3_N_4_. The reason for this phenomenon is that the mechanical properties of *m*-C_3_N_4_ are not excellent compared with the other C_3_N_4_ allotropes and the bulk modulus, shear modulus and Young’s modulus are all smaller than those of other C_3_N_4_ allotropes.

In materials science, ductility is a solid material’s ability to deform under tensile stress. If a material is brittle, when subjected to stress, it will break without significant deformation (strain). Additionally, these material properties are dependent on pressure. Pugh [56] proposed a simple relationship to judge the plastic properties of materials based on their elastic modulus, *i.e.*, *B*/*G*. If the ratio *B*/*G* is larger than 1.75, a material exhibits the ductile property; otherwise, the material is brittle. Moreover, Poisson’s ratio *v* is another criterion for judging the plastic properties of materials [57]. A larger *v* value (*v* > 0.26) for a material indicates ductility, while a smaller *v* value (*v* < 0.26) usually denotes brittleness. At ambient pressure, the ratio *B*/*G* and *v* of *α*-C_3_N_4_, *β*-C_3_N_4_, *d*-ZB-C_3_N_4_, *Cubic*-C_3_N_4_, *Pseudocubic*-C_3_N_4_, *m*-C_3_N_4_ and *t*-C_3_N_4_ are as listed in Table 3. The ratio *B*/*G* and *v* of four C_3_N_4_ allotropes are all less than 1.75 and 0.26, respectively, which indicates that the four C_3_N_4_ allotropes are all brittle. *t*-C_3_N_4_ has the most brittleness, while *β*-C_3_N_4_ has the least brittleness. The pressure dependence of *B*/*G* and Poisson’s ratio *v* are shown in Figure 5c,d, respectively. In Figure 5c,d, the *B*/*G* and *v* of *m*-C_3_N_4_ and *t*-C_3_N_4_ increase with increasing pressure. *m*-C_3_N_4_ is found to change from brittle to ductile at 71 GPa, while *t*-C_3_N_4_ does not change from brittle to ductile in this pressure range.

The elastic anisotropy of a solid is closely related to the possibility of inducing microcracks in materials and can be expressed by the universal anisotropic index (*A*^U^) [58]. The universal anisotropic index is defined as *A*^U^ = 5*G*_V_/*G*_R_ + *B*_V_/*B*_R_-6. The calculated results of universal anisotropic index are also shown in Table 3. The universal anisotropic index of *α*-C_3_N_4_ is only 0.073, which is approximately one-third that of *β*-C_3_N_4_, approximately one-sixth that of *t*-C_3_N_4_, and approximately one-sixteenth that of *m*-C_3_N_4_. Namely, *α*-C_3_N_4_ and *m*-C_3_N_4_ exhibit the smallest and largest elastic anisotropy in *A*^U^, respectively. The pressure dependence of the universal anisotropic index is shown in Figure 5e. The universal anisotropic index of *m*-C_3_N_4_ increases faster than that of *t*-C_3_N_4_. The reason for this phenomenon is that the difference between the value of Voigt and Reuss approximations of shear modulus for *m*-C_3_N_4_ are greater than that of *t*-C_3_N_4_. At 0 GPa (100 GPa), the Voigt approximation values of the shear modulus for *m*-C_3_N_4_ and *t*-C_3_N_4_ are 293.26 GPa (403.14 GPa) and 360.75 GPa (534.66 GPa), respectively. The Reuss approximation values of the shear modulus for *m*-C_3_N_4_ and *t*-C_3_N_4_ are 254.44 GPa (262.45 GPa) and 340.05 GPa (484.94 GPa) at 0 GPa (100 GPa), respectively. The difference between the values of the Voigt and Reuss approximations of the shear modulus for *m*-C_3_N_4_ ranges from 38.8 to 140.7 GPa at 0 GPa and 100 GPa, respectively. Nevertheless, the difference between the value of the Voigt and Reuss approximations of the shear modulus for *t*-C_3_N_4_ only ranges from 20.7 to 49.7 GPa at 0 GPa and 100 GPa, respectively. Thus, the universal anisotropic index *m*-C_3_N_4_ increases faster than that of *t*-C_3_N_4_.

**Figure 6:**
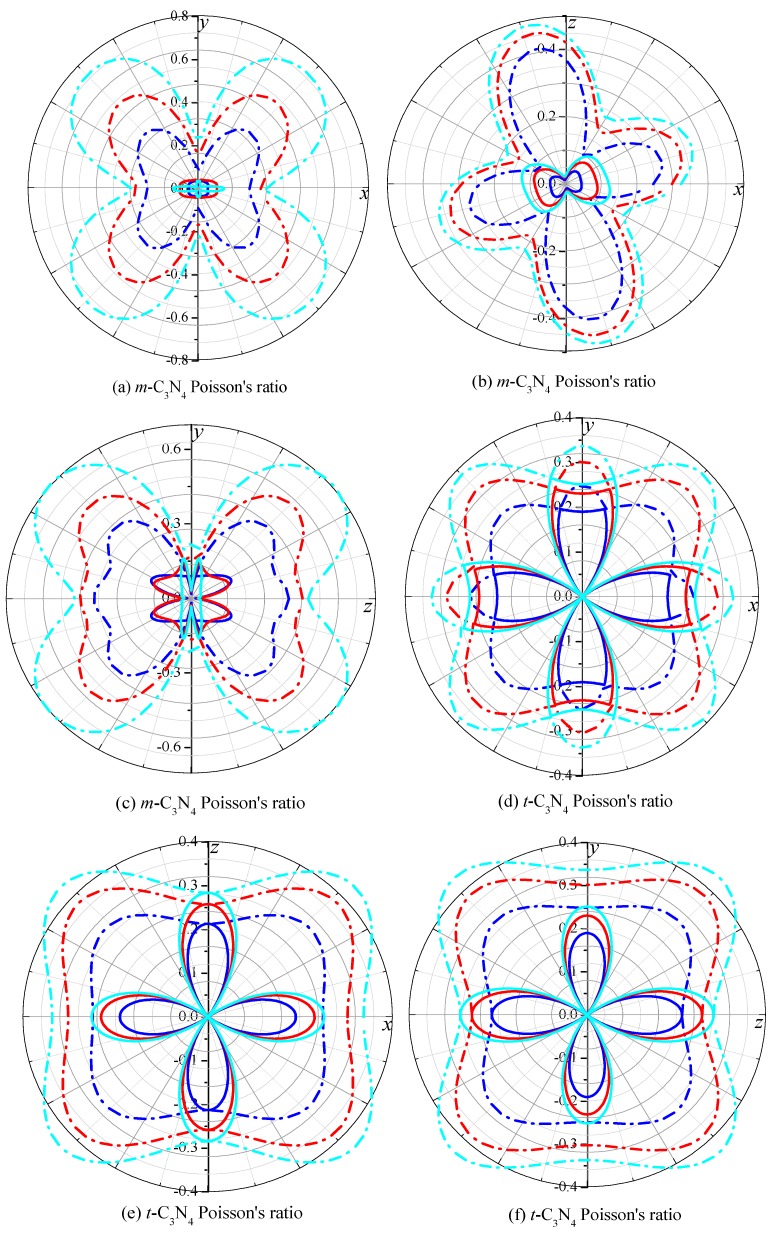
2D representation of Poisson’s ratio in the *xy* plane, *xz* plane and *yz* plane for: *m*-C_3_N_4_ (**a**–**c**); and *t*-C_3_N_4_ (**d**–**f**). The solid line represents the minimum, and the dashed line represents the maximum. The blue, red and cyan lines represent Poisson’s ratio at *p* = 0, 50 and 100 GPa, respectively.

To analyze the anisotropy of *m*-C_3_N_4_ and *t*-C_3_N_4_ more systematically, we will investigate the anisotropy of *m*-C_3_N_4_ and *t*-C_3_N_4_ for Poisson’s ratio, the shear modulus and Young’s modulus by utilizing the ELAM codes [20,59]. The two-dimensional representations of Poisson’s ratio in the *xy* plane, *xz* plane and *yz* plane for *m*-C_3_N_4_ and *t*-C_3_N_4_ are shown in Figure 6. The blue, red and cyan lines represent Poisson’s ratio at 0, 50 and 100 GPa, while the solid line and dash-dot line represent the minimum and maximum values of Poisson’s ratio in the *xy* plane, *xz* plane and *yz* plane, respectively. From Figure 6, it is clear that the anisotropy of Poisson’s ratio for *m*-C_3_N_4_ and *t*-C_3_N_4_ increases with increasing pressure. The maximum value of Poisson’s ratio for *m*-C_3_N_4_ is 0.47, 0.61 and 0.76 at 0, 50 and 100 GPa, while the minimum value of Poisson’s ratio for *m*-C_3_N_4_ is 0.01; the maximum and minimum values of Poisson’s ratio are the same for *m*-C_3_N_4_ in the *xy* plane, *xz* plane and *yz* plane. The maximum value of Poisson’s ratio for *t*-C_3_N_4_ is 0.30, 0.38 and 0.44 at 0, 50 and 100 GPa, respectively, while the minimum value of Poisson’s ratio for *t*-C_3_N_4_ is 0.0; the maximum and minimum values of Poisson’s ratio are the same for *t*-C_3_N_4_ in the *xy* plane, *xz* plane and *yz* plane. The difference between the maximum and minimum values of Poisson’s ratio for *t*-C_3_N_4_ and *m*-C_3_N_4_ shows that *m*-C_3_N_4_ exhibits greater anisotropy with respect to Poisson’s ratio.

**Figure 7:**
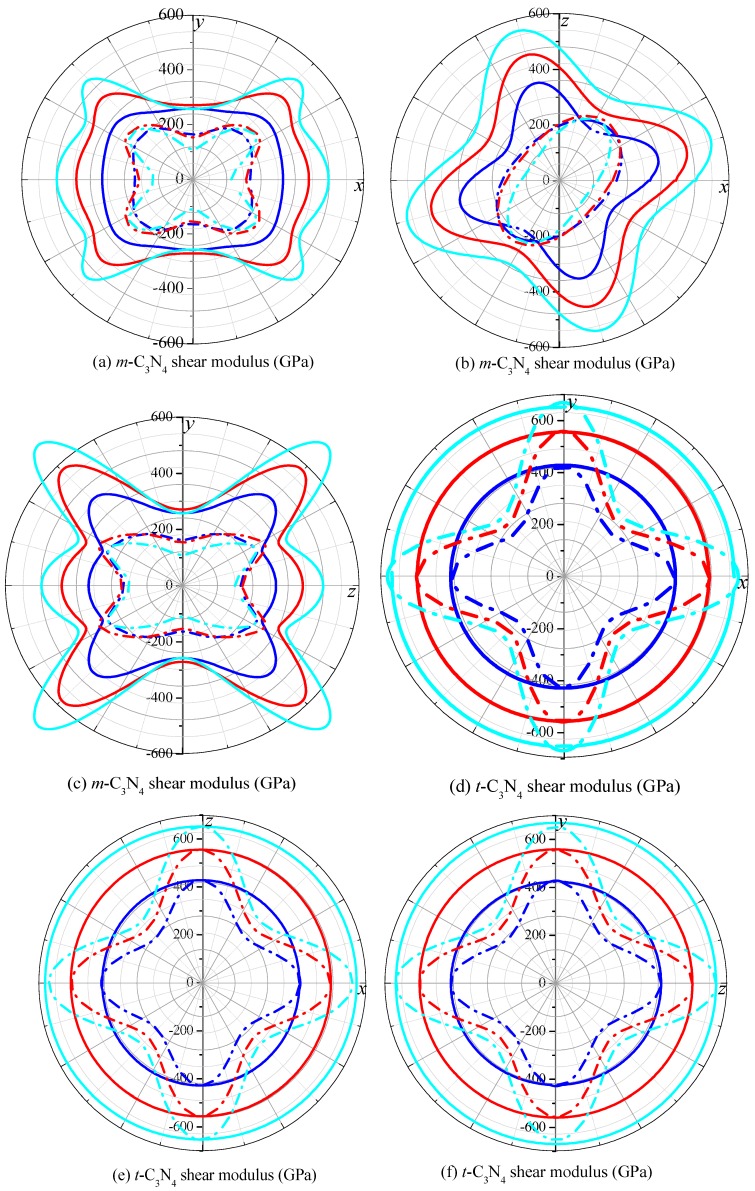
2D representation of shear modulus in the *xy* plane, *xz* plane and *yz* plane for: *m*-C_3_N_4_ (**a**–**c**); and *t*-C_3_N_4_ (**d**–**f**).

The 2D representations of the shear modulus in the *xy* plane, *xz* plane and *yz* plane for *m*-C_3_N_4_ and *t*-C_3_N_4_ are illustrated in Figure 7. For *m*-C_3_N_4_ in Figure 7a–c, the maximum value of the shear modulus occurs in the deviation from the *x* axis or *y* axis of approximately 45 degrees in the *xy* plane or *yz* plane, respectively. The maximum value of the shear modulus occurs in the deviation from the *x* axis or *z* axis of approximately 15 degrees in the *xz* plane. Moreover, the minimum value of the shear modulus occurs along the *y* axis in the *xy* plane and *yz* plane, respectively. The maximum and the minimum values of *m*-C_3_N_4_ are 163 GPa and 455 GPa at ambient pressure, respectively, and the ratio *G*_max_/*G*_min_ = 2.79. At the same time, the maximum and minimum values of *m*-C_3_N_4_ are 153 GPa (110 GPa) and 611 GPa (738 GPa), respectively, at 50 GPa (100 GPa); the ratio *G*_max_/*G*_min_ = 3.99 at *p* = 50 GPa, and the ratio *G*_max_/*G*_min_ = 6.71 at *p* = 100 GPa. The anisotropy of *m*-C_3_N_4_ increases with increasing pressure. The average shear modulus of *m*-C_3_N_4_ is 266 GPa, 312 GPa and 328 GPa, respectively. For *t*-C_3_N_4_ in Figure 7d–f, the maximum value of the shear modulus for *t*-C_3_N_4_ in the *xy* plane, *xz* plane and *yz* plane appears along the coordinate axis, while the minimum value of the shear modulus for *t*-C_3_N_4_ in the *xy* plane, *xz* plane and *yz* plane appears in the deviation from the coordinate axis of approximately 45 degrees. The maximum and minimum values of the shear modulus for *t*-C_3_N_4_ are 245 GPa, 291 GPa, 323 GPa and 428 GPa, 559 GPa, 669 GPa at 0 GPa, 50 GPa, 100 GPa, respectively. The ratio *G*_max_/*G*_min_ = 1.75 at *p* = 0 GPa, the ratio *G*_max_/*G*_min_ = 1.92 at *p* = 50 GPa, and the ratio *G*_max_/*G*_min_ = 2.07 at *p* = 100 GPa. It is clear that the ratio *G*_max_/*G*_min_ for *t*-C_3_N_4_ is much smaller than that for *m*-C_3_N_4_. In other words, *m*-C_3_N_4_ exhibits greater anisotropy than *t*-C_3_N_4_. This agrees well with our previous prediction of anisotropy with respect to the universal anisotropic index and Poisson’s ratio.

**Figure 8:**
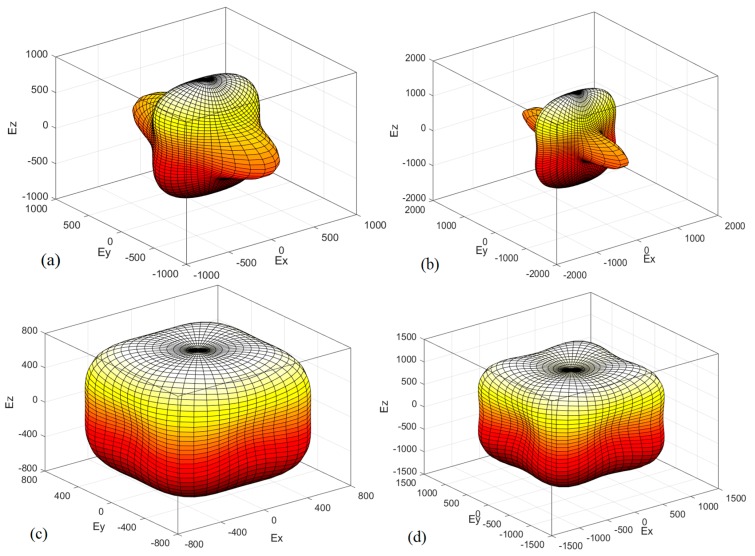
The directional dependence of Young’s modulus for: *m*-C_3_N_4_ (**a**,**b**); and *t*-C_3_N_4_ (**c**,**d**).

**Figure 9:**
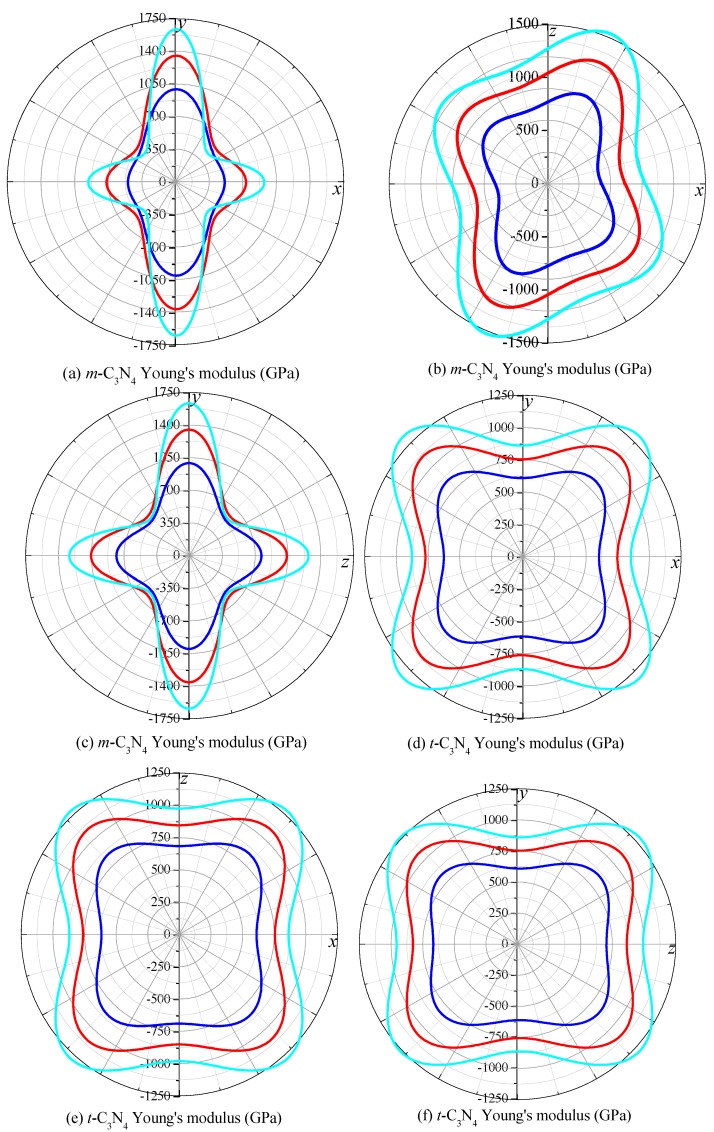
2D representation of Young’s modulus in the *xy* plane, *xz* plane and *yz* plane for: *m*-C_3_N_4_ (**a**–**c**); and *t*-C_3_N_4_ (**d**–**f**). The blue, red and cyan lines represent Poisson’s ratio at *p* = 0, 50 and 100 GPa, respectively.

As a valid method to describe the elastic anisotropic behavior of a crystal completely, the 3D surface constructions of the directional dependences of reciprocals of Young’s modulus are practically useful. The results are shown in Figure 8 for Young’s modulus. For an isotropic system, the 3D directional dependence would exhibit a spherical shape, while the deviation degree from the spherical shape reflects the content of anisotropy [60]. In Figure 8a,c, the 3D shape of Young’s modulus shows that *m*-C_3_N_4_ exhibits greater anisotropy than *t*-C_3_N_4_. As the pressure increases, the anisotropy of Young’s modulus for *m*-C_3_N_4_ and *t*-C_3_N_4_ increases, but *m*-C_3_N_4_ still exhibits greater anisotropy than *t*-C_3_N_4_. To analyze the anisotropy of *m*-C_3_N_4_ and *t*-C_3_N_4_ in detail, the 2D representations of Young’s modulus in the *xy* plane, *xz* plane and *yz* plane for *m*-C_3_N_4_ and *t*-C_3_N_4_ are depicted in Figure 9. From Figure 9, it is clear that *m*-C_3_N_4_ has a larger anisotropy and that the anisotropy will become larger with increasing pressure.

Young’s modulus of *t*-C_3_N_4_ has the same value in different planes, while that of *m*-C_3_N_4_ has different values in different planes. For example, at ambient pressure, the maximum and minimum values of Young’s modulus for *t*-C_3_N_4_ are 928 GPa and 612 GPa in the *xy* plane, *xz* plane and *yz* plane, while at *p* = 100 GPa, they are 1510 GPa and 864 GPa, respectively. However, the maximum value of Young’s modulus is 996 GPa in the *xy* plane and *yz* plane for *m*-C_3_N_4_, but in the *xz* plane, it is 995 GPa, and the minimum value is always 476 GPa. At 100 GPa, the difference reaches a larger degree; in the *xy* plane and *yz* plane, the maximum value of Young’s modulus for *m*-C_3_N_4_ is 1638 GPa, while the maximum value is 1634 GPa. This also proves that *m*-C_3_N_4_ has a larger anisotropy from the other side.

### 3.3. Electronic Structures

**Figure 10:**
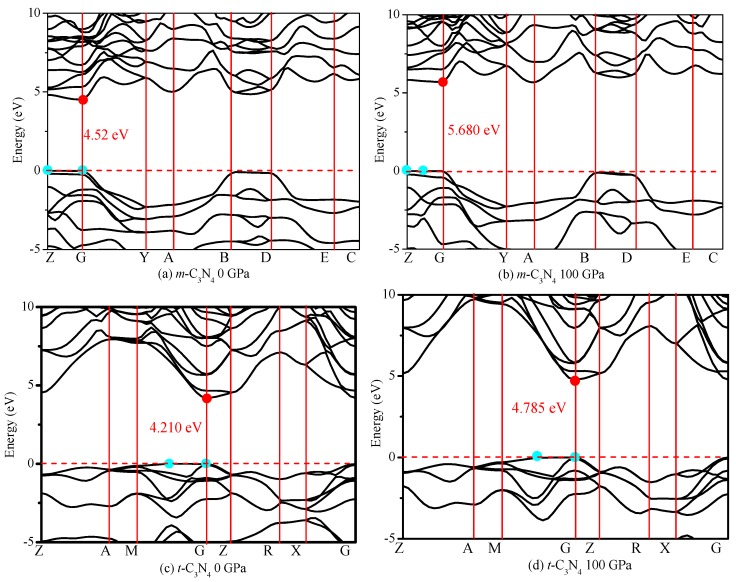
Electronic band structure of: *m*-C_3_N_4_ (**a**,**b**); and *t*-C_3_N_4_ (**c**,**d**) with HSE06.

Band theory is one of the most stringent tests of the physics of semiconductors. For example, silicon, calcite and copper all contain similar densities of electrons, but they have different physical properties, all inexplicable without quantum mechanics [61]. Thus, it is necessary to understand the band structure and density of states. The band structures and density of states of *m*-C_3_N_4_ and *m*-C_3_N_4_ at different pressures are shown in Figure 10. The band structure calculations show that *m*-C_3_N_4_ is a quasi-direct band gap semiconductor, with a band gap of 4.52 eV (see Figure 10a), and at 100 GPa, *m*-C_3_N_4_ remains a quasi-direct band gap semiconductor, with a band gap of 5.68 eV. *t*-C_3_N_4_ has a quasi-direct band gap of 4.21 eV at (0.322 0.322 0.0) along the M−Γ direction and Γ point, while the direct band gap is 4.22 eV at the Γ point. At 100 GPa, *t*-C_3_N_4_ has a quasi-direct band gap of 4.79 eV at (0.322 0.322 0.0) along the M−Γ direction and Γ point, while the direct band gap is 4.81 eV at the Γ point. At 0 GPa, the valence band maximum (VBM) of *m*-C_3_N_4_ is located at the Z point, the energy of VBM near the Fermi level of *m*-C_3_N_4_ is 10.37 eV, and the energy of the Z point near the Fermi level is 10.39 eV; thus, *m*-C_3_N_4_ is a quasi-direct band gap semiconductor. At 100 GPa, the Fermi level of *m*-C_3_N_4_ increases to 14.04 eV, and the energy of the Z point near the Fermi level of *m*-C_3_N_4_ is 14.02 eV. The VBM of *m*-C_3_N_4_ is located at the point along the Z and Γ directions; its energy is 14.04 eV. The conduction band minimum (CBM) is at the Γ point for *m*-C_3_N_4_ at 0 and 100 GPa. The energy of CBM is 14.91 and 19.72 eV. At 0 GPa, the Fermi level of *m*-C_3_N_4_ is 10.39 eV, which is slightly smaller than that of *t*-C_3_N_4_ (10.52 eV). For *t*-C_3_N_4_, the CBM is at the Γ point for *t*-C_3_N_4_ at 0 and 100 GPa; the energy of CBM is 14.73 and 18.82 eV, respectively. The VBM of *t*-C_3_N_4_ is located at the point along the M and Γ directions; the energy is 10.52 and 14.03 eV, respectively. Moreover, the energy of the Γ point near the Fermi level of *t*-C_3_N_4_ is 10.51 eV and 14.01 eV at 0 and 100 GPa, respectively. Thus, *t*-C_3_N_4_ is a quasi-direct band gap semiconductor. Interestingly, the band gaps of *m*-C_3_N_4_ and *t*-C_3_N_4_ both increase with increasing pressure. At 100 GPa, *m*-C_3_N_4_ increases by 25.61%, and *t*-C_3_N_4_ increases by 13.66% compared with that at 0 GPa.

## 4. Conclusions

In conclusion, we have predicted two novel C_3_N_4_ allotropes, *i.e.*, *m*-C_3_N_4_ and *t*-C_3_N_4_, with space groups *Cm* and *I*-42*m*, which are both mechanically and dynamically stable up to at least 100 GPa. The bulk modulus of *t*-C_3_N_4_ is 375 GPa, which is slightly larger than that of *c*-BN, while the bulk modulus of *m*-C_3_N_4_ is slightly smaller than that of *c*-BN. The hardness of *t*-C_3_N_4_ is larger than that of *c*-BN, thereby making it a superhard material with potential technological and industrial applications. The ratio *B*/*G* and *v* of the two novel C_3_N_4_ phases are both less than 1.75 and 0.26, respectively, which indicates that the two novel C_3_N_4_ allotropes are both brittle. The *B*/*G* and *v* of *m*-C_3_N_4_ and *t*-C_3_N_4_ increase with increasing pressure. *m*-C_3_N_4_ is found to change from being brittle to ductile at 71 GPa, while *t*-C_3_N_4_ does not change from being brittle to ductile in this pressure range. The elastic anisotropy calculations show that *m*-C_3_N_4_ and *t*-C_3_N_4_ both exhibit large anisotropy with respect to Poisson’s ratio, the shear modulus and Young’s modulus and universal anisotropic index. The band structure calculations show that *m*-C_3_N_4_ and *t*-C_3_N_4_ are a quasi-direct-band-gap semiconductor and a quasi-direct-band-gap semiconductor, respectively. Moreover, the band gaps of *m*-C_3_N_4_ and *t*-C_3_N_4_ continue to be a quasi-direct band-gap and quasi-direct band gap at 100 GPa, respectively. The band gaps of *m*-C_3_N_4_ and *t*-C_3_N_4_ are 4.522 and 4.210 eV, respectively, and these materials are both wide-band-gap semiconductors. Due to their quasi-direct band gaps, they are attractive for luminescent device applications.

## Figures and Tables

**Table 1:** Calculated lattice parameters (Å) of *m*-C_3_N_4_, *t*-C_3_N_4_, *d*-ZB-C_3_N_4_, *Cubic*-C_3_N_4_, *Pseudocubic*-C_3_N_4_ and *g*-C_3_N_4_.

Materials	This Work			Other Works
	*a*	*b*	*c*	*a*, *b*, *c*
*α*-C_3_N_4_	6.512	–	4.742	*a* = 6.489, *c* = 4.729 ^a^; *a* = 6.425, *c* = 4.715 ^b^; *a* = 6.467, *c* = 4.710 ^c^; *a* = 6.453, *c* = 4.699 ^d^; *a* = 6.47, *c* = 4.71 ^e^
*β*-C_3_N_4_	6.449	–	2.422	*a* = 6.426, *c* = 2.418 ^a^; *a* = 6.419, *c* = 2.425 ^b^; *a* = 6.402, *c* = 2.404 ^c^; *a* = 6.394, *c* = 2.397 ^d^; *a* = 6.40, *c* = 2.40 ^e^
*d*-ZB-C_3_N_4_	3.456	–	–	*a* = 3.455 ^f^; *a* = 3.52 ^e^; *a* = 3.43 ^g^
*cubic*-C_3_N_4_	5.398	–	–	*a* = 5.395–5.444 ^h^; *a* = 5.40 ^e^
*pseudocubic*-C_3_N_4_	3.456	–	–	*a* = 3.41–3.44 ^h^
*g*-C_3_N_4_	4.791	–	6.769	*a* = 4.74, 6.72 ^e^
*m*-C_3_N_4_	8.032	2.418	6.246	–
*t*-C_3_N_4_	3.483	–	6.933	–

^a^ Reference [37]; ^b^ Reference [38]; ^c^ Reference [23]; ^d^ Reference [39]; ^e^ Reference [40]; ^f^ Reference [41]; ^g^ Reference [22]; ^h^ Reference [42].

**Table 2:** The calculated elastic constants (GPa) of *m*-C_3_N_4_, *t*-C_3_N_4_, *d*-ZB-C_3_N_4_, *cubic*-C_3_N_4_, *pseudocubic*-C_3_N_4_ and *cubic*-BN.

Materials	*C*_11_	*C*_22_	*C*_33_	*C*_44_	*C*_55_	*C*_66_	*C*_12_	*C*_13_	*C*_23_	*C*_15_	*C*_25_	*C*_35_	*C*_46_
*α*-C_3_N_4_	848	–	906	319	–	335	179	131	–	−27	–	–	27
Reference [37]	851	–	906	326	–	334	183	129	–	–	–	–	–
*β*-C_3_N_4_	852	–	1150	286	–	312	228	111	–	–	–	–	–
Reference [37]	833	–	1049	289	–	287	259	110	–	–	–	–	–
Reference [22]	834	–	1120	305	–	–	279	138	–	–	–	–	–
*d*-ZB-C_3_N_4_	791	–	–	443	–	–	184	–	–	–	–	–	–
Reference [41]	794	–	–	431	–	–	184	–	–	–	–	–	–
*Cubic*-C_3_N_4_	889	–	–	518	–	–	309	–	–	–	–	–	–
Reference [26]	861	–		469	–	–	300	–	–	–	–	–	–
*Pseudocubic*-C_3_N_4_	790	–	792	445	–	444	188	187	–	–	–	–	–
Reference [37]	804	–	805	439	–	439	183	183	–	–	–	–	–
*m*-C_3_N_4_	564	1002	859	209	331	213	51	195	36	−57	6	60	48
*t*-C_3_N_4_	702	–	767	428	–	424	212	195	–	–	–	–	–
*Cubic*-BN	823	–	–	479	–	–	185	–	–	–	–	–	–
Reference [50]-Exp.	820	–	–	480	–	–	190	–	–	–	–	–	–

**Table 3:** The calculated elastic modulus (GPa), *B*/*G*, hardness *Hv* (GPa) and the universal anisotropic index of *m*-C_3_N_4_, *t*-C_3_N_4_, *d*-ZB-C_3_N_4_, *cubic*-C_3_N_4_, *pseudocubic*-C_3_N_4_ and *cubic*-BN.

Materials	*B*_V_	*B*_R_	*G*_V_	*G*_R_	*B*_H_	*G*_H_	*B*/*G*	*E*	*v*	*Hv*	*A*^U^
*α*-C_3_N_4_	386.83	386.82	338.65	335.09	387	337	1.15	784	0.16	78	0.053
Reference [48]	387.60	387.60	341.85	338.96	388	340	1.14	790	0.16	82	0.043
*β*-C_3_N_4_	406.11	405.57	330.77	320.96	406	326	1.25	772	0.18	78	0.154
Reference [48]	408.21	407.84	322.09	311.12	408	317	1.29	755	0.19	63, 85 ^a^	0.176
*d*-ZB-C_3_N_4_	386.59	386.59	387.23	374.16	387	381	1.02	861	0.13	81	0.175
Reference [51]	–	–	–	–	387	375	1.03	850	0.13	63	–
*Cubic*-C_3_N_4_	502.48	502.48	426.82	394.18	502	411	1.22	969	0.18	88	0.414
Reference [48]	–	–	–	–	496	401	1.24	948	0.18	90, 87 ^b^	–
*Pseudocubic*-C_3_N_4_	388.21	388.21	387.66	373.98	388	381	1.02	861	0.13	81	0.183
Reference [48]	390.02	390.02	387.76	376.88	390	382	1.02	864	0.13	70, 80 ^c^	0.144
*m*-C_3_N_4_	332.22	320.80	293.26	254.44	327	274	1.19	643	0.17	37	0.798
*t*-C_3_N_4_	374.81	374.47	360.75	340.05	375	350	1.07	801	0.14	80	0.305
*c*-BN	397.43	397.43	414.90	398.88	397	407	0.98	910	0.12	63	0.201
-	–	–	–	–	400 ^d^	–	–	–	–	66 ^e^, 64 ^f^	–

^a^ Reference [52]; ^b^ Reference [51]; ^c^ Reference [53]; ^d^ Reference [50]; ^e^ Reference [54]; ^f^ Reference [55].
